# A Tool for Prioritizing Livestock Disease Threats to Scotland

**DOI:** 10.3389/fvets.2020.00223

**Published:** 2020-04-24

**Authors:** Paul R. Bessell, Harriet K. Auty, Helen Roberts, Iain J. McKendrick, B. Mark de C. Bronsvoort, Lisa A. Boden

**Affiliations:** ^1^The Roslin Institute, The University of Edinburgh, Edinburgh, United Kingdom; ^2^Epidemiology Research Unit, SRUC, An Lòchran, Inverness Campus, Inverness, United Kingdom; ^3^Institute of Biodiversity, Animal Health and Comparative Medicine, College of Medical, Veterinary and Life Sciences, University of Glasgow, Glasgow, United Kingdom; ^4^Exotic Disease Control Team, Defra, London, United Kingdom; ^5^Biomathematics and Statistics Scotland, Edinburgh, United Kingdom; ^6^Global Academy of Agriculture and Food Security, The Royal (Dick) School of Veterinary Studies and The Roslin Institute, The University of Edinburgh, Easter Bush Campus, Midlothian, United Kingdom

**Keywords:** livestock, disease, introduction, risk, horizon scanning

## Abstract

There are a number of disease threats to the livestock of Scotland that are not presently believed to be circulating in the UK. Here, we present the development of a tool for prioritizing resources for livestock disease threats to Scotland by combining a semi-quantitative model of the chance of introduction of different diseases with a semi-quantitative model of disease impact. Eighteen key diseases were identified and then input into a model framework to produce a semi-quantitative estimate of disease priorities. We estimate this through a model of the potential impacts of the infectious diseases in Scotland that is interpreted alongside a pre-existing generic risk assessment model of the risks of incursion of the diseases. The impact estimates are based on key metrics which influence the practical impact of disease. Metrics included are the rate of spread, the disease mitigation factors, impacts on animal welfare and production, the human health risks and the impacts on wider society. These quantities were adjusted for the size of the Scottish livestock population and were weighted using published scores. Of the 18 livestock diseases included, the model identifies highly pathogenic avian influenza, foot and mouth disease in cattle and bluetongue virus in sheep as having the greatest priority in terms of the combination of chance of introduction and disease impact. Disregarding the weighting for livestock populations and comparing equally between industry sectors, the results demonstrate that Newcastle disease and highly pathogenic avian influenza generally have the greatest potential impact. This model provides valuable information for the veterinary and livestock industries in prioritizing resources in the face of many disease threats. The system can easily be adjusted as disease situations evolve.

## Introduction

Since 2000 there have been incursions of high profile diseases such as Foot and Mouth Disease (FMD), Classical Swine Fever (CSF) and Bluetongue virus (BTV) in the United Kingdom (UK) that have caused large outbreaks with high impacts, resulting in high costs (1–3). The UK has also had a number of incursions of highly pathogenic avian influenza (HPAI) that resulted in smaller outbreaks, but with the potential for great impact should HPAI become established (4, 5). There is an ongoing outbreak of African Swine Fever (ASF) in Europe (6) and there have been outbreaks of lumpy skin disease, sheep pox, and peste des petits ruminants in the Balkans (7–10). Policymakers can take actions to reduce the chance of incursion, or to prepare for potential disease outbreaks, but have to prioritize between different pathogen threats. Assessing the risks posed by such threats requires consideration of both the chance of incursion and the impact following the arrival of the disease.

The potential impacts of some diseases have been assessed using mathematical models of disease spread (11–14) but comparable mathematical modeling frameworks are not available for all diseases. In the absence of a single consistent modeling framework the impacts of an infectious disease can be evaluated with respect to a number of criteria. These include the potential extent of spread of the disease in terms of the likely numbers of animals that may become infected. This effect is offset by the mitigating factors that may exist such as the availability and effectiveness of vaccines, the seasonality of the pathogen, and whether there are potential reservoirs of infection in vectors and wildlife. A disease outbreak will have direct impacts on animal health, welfare and productivity as well as potential secondary effects on human health. There are also indirect impacts on international trade and impacts on society as a whole. This includes both the costs of controlling the disease and wider impacts on rural economies as seen during the 2001 FMD outbreak (1).

Estimating the chance of incursion of a particular disease is important in prioritizing the threat from that disease. Defra has developed a tool for assessing and assigning a risk ranking on the incursion of different diseases (15). The tool combines the current known global distribution of diseases with data on the likelihood of different pathways of introduction, the products that are traded and existing risk mitigation measures that are in place. The diseases are classified according to the EFSA risk level classification scale shown in Table 1, which provides a consistent mapping from the estimated levels of risk to a scale of probabilities, which we will interpret as being the chances of an incursion leading to an appreciable outbreak.

**Table 1:** The risk classification scale used in the Defra risk of incursion tool (15, 16).

**Probability**	**Score**	**Definition from EFSA**	**Expanded description**
Negligible	0–10	Event is so rare that is does not merit consideration	The chance of the event occurring is so small it does not merit consideration in practical terms; it is not expected to happen for many years, if at all
Very low	10–20	Event is very rare but cannot be excluded	The event is not expected to occur (very rare) in the next few years but it is possible
Low	20–30	Event is rare but does occur	The event may occur occasionally (rare) but could occur in the next few years
Medium	30–40	Event occurs regularly	The event is possible within the next year
High	>40	Event occurs very often	The event is expected to occur within the next year

Two methods have been developed for comparing potential impacts directly and consistently between diseases. Defra has developed the Disease briefing, Decision support, Ranking and Risk assessment (D2R2) database (17) and the DISCONTOOLS Project that aims to identify knowledge gaps in diseases (18). Whilst the methods underlying both methods are different, both are essentially based on expert elicitation.

The aims of this paper are to demonstrate the value of a model to prioritize disease threats to Scotland based on estimates of their chance of incursion (hereafter denoted by *r*) and potential impact following introduction. Such a model can be used by industry and government veterinary agencies to prioritize surveillance and preparedness resources. The tool will use data from DISCONTOOLS and D2R2 to derive an index of disease impact. This will then be combined with the chance of incursion based on the risk of incursion scores defined in Roberts et al. (15) to develop a risk matrix capturing variability across the two contributory axes, namely chance of introduction and disease impact. To compare our measure to the impact as perceived by the scientific research community, we examine the estimated impact of the diseases against a metric which seeks to measure the extent of scientific research into each pathogen.

## Materials and Methods

In discussion with the Animal Health and Welfare Department (AHW) at the Scottish Government, a list of 18 priority diseases was identified (Table 2). Rather than a static list the priority diseases were refined over a period of years between 2012 and 2019 as new threats emerged and the priorities of the AHW department changed, for example, in response to the emergence of lumpy skin disease in the Balkans. Some of the diseases affect multiple host species; these were treated separately when modeling the impact in different species. Both low pathogenic and high pathogenic avian influenza were included, due to their differing impacts and epidemiology. Bluetongue virus (BTV) has clinical presentations that are both highly pathogenic and less pathogenic, here we consider a more highly pathogenic presentation (19). Caprine diseases were not included because the population of goats in Scotland is small (20).

**Table 2:** Diseases included in these analyses.

**Disease**	**Domestic species affected**	**Zoonotic**	**History of occurrence^a^**	**Mode of transmission**
Brucellosis (*B. abortus*)	Cattle	Yes	2003 (Scotland) 2004 (England)	Direct, indirect contact—fetal material, uterine discharges, milk
Enzootic bovine leukosis (EBL)	Cattle	No	1999 (UK)	Direct, indirect, vertical
Lumpy skin disease (LSD)	Cattle	No	Never	Biting flies, mosquitoes
Bluetongue Virus (BTV)	Cattle, sheep	No	2007 (England)	Vector—Culicoides
Foot and mouth disease (FMD)	Cattle, sheep, pigs	No	2007 (England)	Direct, indirect contact
African Swine Fever (ASF)	Pigs	No	Never	Direct contact, vector–exotic soft ticks
Aujeszky's Disease	Pigs	No	Eradicated 1989	Direct, indirect contact
Classical Swine Fever (CSF)	Pigs	No	2000 (England)	Direct, indirect contact
Swine Vesicular Disease (SVD)	Pigs	No	Eradicated 1982	Direct, indirect contact
Porcine Epidemic Diarrhea (PED)	Pigs	No	Eradicated 1982	Fecal-oral, fomites, germplasm, airborne
Sheep pox	Sheep	No	Eradicated 1866	Direct contact
Peste des petits ruminants (PPR)	Sheep	No	Never	Direct, indirect contact
Low Pathogenic Avian Influenza (LPAI)	Poultry	Yes	2018 (Scotland)	Direct, indirect contact, wild birds
Highly Pathogenic Avian Influenza (HPAI)	Poultry	Yes	2018 (Scotland)	Direct, indirect contact, wild birds
Newcastle Disease (ND)	Poultry	Yes	2006	Direct, indirect contact, wild birds
African Horse Sickness (AHS)	Equines	No	Never	Vector–Culicoides
Equine Infectious Anemia (EIA)	Equines	No	2012 (England)	Mechanical vector–Tabanids
West Nile Virus (WNV)	Equines	Yes	Never	Vector–Mosquitoes

* *If there has been an outbreak in Scotland this is recorded, otherwise the most recent outbreak elsewhere in GB is given*.

### Livestock Populations

To allow for differences in the size of the population and the values of animals of different species and ages, species population data were taken from the Scottish agricultural census from June 2018 (21). The horse population is an estimate from Horse Scotland (22). The Scottish Agricultural census breaks down the animals of each species to different age groups and production classifications (e.g., dairy vs. beef). Each age group and production classification has a value for livestock grazing comparison units based on the Defra Farm Business Survey and cited in Nix (23). By mapping the data from the Scottish Agricultural census to livestock units, we calculate a total value in terms of livestock units for the population of each species in Scotland and in Table 3 we present the mean number of livestock units per head of each species. We then take the square root of these population livestock units as the transformed population for livestock species *i* (${Pop}_{i}^{T}$) (Table 3). In so doing, we are up-weighting the relative importance of smaller populations when evaluating overall impact. This is reasonable, since we believe that it is unlikely that impact will increase *pro-rata* to the population size.

**Table 3:** Populations of livestock in Scotland transformed by the number of livestock units assigned to that species by Nix (23).

**Species**	**Population**	**Population livestock units**	**Mean livestock units per head**	**Transformed population (${Pop}_{i}^{T}$)**
Cattle	1,755,318	1,125,158.84	0.641	1,060.7
Horses	100,000	80,000	0.800	282.8
Pigs	316,736	60,179.84	0.190	245.3
Poultry	14,541,621	101,791.347	0.007	319.0
Sheep	6,593,410	402,198.01	0.061	634.2

### Disease Impact

The source of the estimates of disease impact was the DISCONTOOLS project, informed by the Defra D2R2 system. Impact is scored based on 6 sub-categories in three broad categories:

1. Extent of spread:
  - Rate of spread.
  - Mitigation factors including the availability of effective vaccines, wildlife reservoirs, vector reservoirs and opportunities to control the disease through biosecurity and through movement bans.
2. Health and welfare:
  - Animal welfare (including morbidity) and animal mortality.
  - Human health.
3. Indirect impacts
  - Wider society to include the impacts of the disease on restrictions to human activities, the industry sector, and government finances.
  - International trade.

The parameters that were derived from DISCONTOOLS and D2R2 (17, 18) are described in Tables S1–S3.

The metrics in Tables S1–S3 are combined in a single impact model for disease *d* in species *i*:

$$\begin{array}{l} r_{di}=Pop_{i}^{T}\beta_{di}p_{di}\left(a_{di}+h_{di}\right)c_{di}t_{di} \end{array}$$

In this equation, $Pop_{i}^{T}\beta_{di}p_{di}$ scales the impact with respect to the extent of spread of the disease in the specific transformed population; β_*di*_ quantifies the potential for spread and *p*_*di*_ summarizes and adjusts for the impact of mitigation measures on the potential for spread. The second block of terms, *a*_*di*_, *h*_*di*_, sum to give the direct impact score in terms of animal morbidity and mortality and human health, respectively. All the terms contribute multiplicatively, with the exception of the direct impacts on animal morbidity and mortality and human health *a*_*di*_, *h*_*di*_, which each make an independent additive contribution to the impact score. This is to reflect the discreet impacts of disease on human and livestock populations and to scale the sum of these to be between 0 and 2, recognizing that most of the diseases have no human health impact and therefore give rise to a factor taking values between 0 and 1. In this way, the effect of direct impacts is consistent with the effects of indirect impacts on society and trade *c*_*di*_, *t*_*di*_ that are each also scaled to be between 0 and 1.

The corresponding estimate of impact in species *i*, when we do not adjust for the livestock population is:

$$\begin{array}{l} r_{di}^{*}=\beta_{di}\left(1-p_{di}\right)\left(a_{di}+h_{di}\;\right)c_{di}t_{di}. \end{array}$$

We will illustrate the approach to quantifying individual terms by considering the potential for spread (β_*d*_) of disease *d*. This is estimated as the sum of scores over the set of relevant determining factors in species *j* (*s*_*j*_) (Table S1) normalized relative to the maximum possible sum of factor scores:

$$\begin{array}{l} \beta_{d}=\frac{\sum_{j}s_{jd}}{\sum_{j}\max\left(s_{j}\right)}. \end{array}$$

A similar formulation based on a weighted sum of scores was used for each of the other parameters (Tables S1–S3): effect of mitigation factors (*p*_*di*_), animal health factors (*a*_*di*_), human health factors (*h*_*di*_), wider society (*c*_*di*_), international trade (*t*_*di*_), each being calculated and scaled using the specific determining factors for that metric, for that disease and species. In the equation we multiply by (1 − *p*_*di*_) because values of 1 for *p*_*d*_ correspond to strong mitigation and values of 0 to no mitigation. An example of the calculation of impact is given in Supplementary Information S2.

### Chance of Incursion

The chance of incursion of each pathogen is taken from the risk of incursion tool (15) (update from March 2019). This takes into account the current global distribution of the diseases, possible routes of entry (including migrating birds) and disease mitigations that are in place in the country or region of origin.

## Results

**Figure 1:**
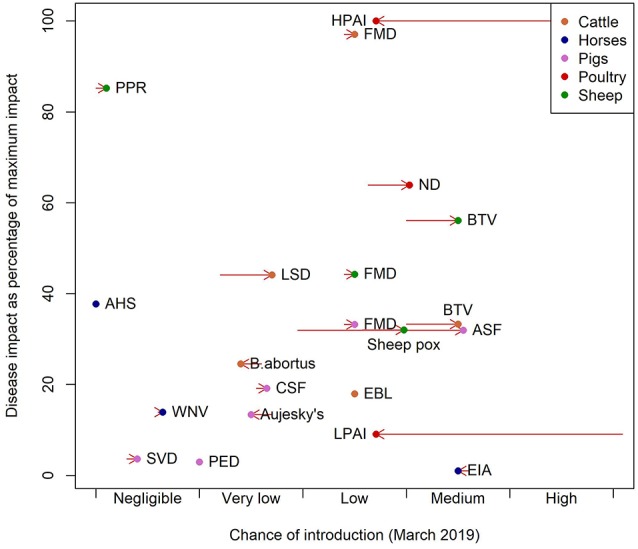
Chance of introduction against impact as of March 2019. Impact is presented as the percentage of the impact of the disease with greatest impact. The arrows represent the change in the chance of introduction from the position in March 2017 to chance of introduction in March 2019.

Examining the pattern of estimated potential impacts relative to the specific chance of incursion (Figure 1), diseases can be categorized and hence prioritized. HPAI, BTV in sheep, FMD in cattle and ND are diseases with high impact and a low or medium chance of introduction (top right hand corner of Figure 1). BTV in cattle and ASF both have medium chances of introduction, but slightly lower impact and EIA a very low impact (Figure 1). PPR has a negligible chance of introduction but is a disease with potentially high impact (Figure 1). The decomposition of these scores is provided in the (Figures S1–S4).

Comparing the changes in risk between March 2017 and March 2019 shows a large decrease in the relative importance of avian influenzas and increases in risks of ASF, BTV, ND, sheep pox and LSD (Figure 1). These are driven by the change in their chance of introduction driven by changes in the distribution of the pathogens in Europe.

**Figure 2:**
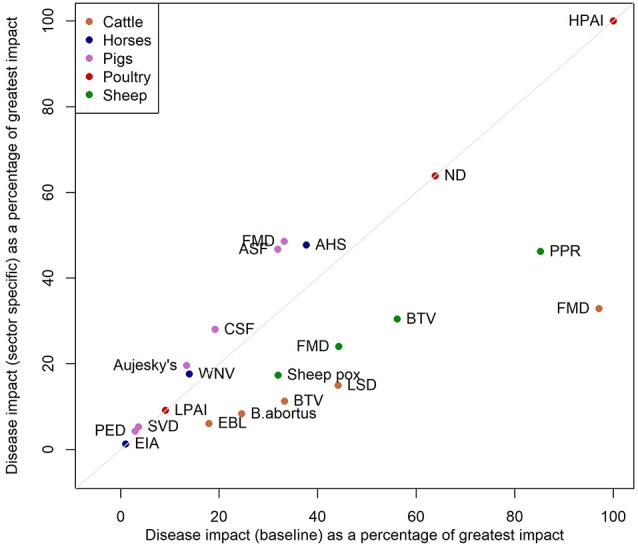
Scatterplot of the impacts from the baseline model against the impacts from a model where we do not include the livestock population sizes in the model.

A sensitivity analysis in which the indirect impact scores (impacts on wider society and international trade) are included additively rather than multiplicatively results in BTV in sheep becoming the most impactful disease. This high impact is driven by the high potential for spread of the disease (Figure S5). Disregarding the size of livestock population so that impacts are considered irrespective of sector, leads to the relative impact of cattle and sheep diseases reducing and the impact in pigs and poultry diseases increasing (Figure 2).

## Discussion

This framework provides a novel way to combine and interpret independent metrics of animal disease impacts. The selected metrics of disease impact were similar in both DISCONTOOLS (18) and D2R2 (17) and were loosely classified as disease spread, impacts on animal and human health and indirect impacts on wider society and international trade. The methodology was implemented over a range of diseases, selected by discussion with policy-makers because the diseases are exotic to Scotland (most are notifiable) and pose a potential threat. Further diseases can be bought in as the model is further developed.

The diseases with the highest overall impact (Figure 1) are cattle FMD, PPR, HPAI, ND, and sheep BTV, but their high impact scores are driven by different factors. In the case of FMD in cattle the indirect factors are key and for HPAI and ND the impacts are driven by the direct factors. For BTV the main driver is the large potential extent of spread due to the fact that it is transmitted by midge vectors (Supplementary Information S1). The impact of BTV is further impacted by the wide range of strains which affects the potential severity of infection and the potential to control disease through vaccination. The purpose of this approach is to summarize disease properties succinctly and effectively: some diseases have high impact in just one area. For example in the case of WNV the impact is predominantly on human and animal health, but WNV has low potential for spread and low indirect impacts and so is assigned a lower overall impact score. Cattle diseases: FMD, BTV, LSD, and brucellosis (*B. abortus*), had the greatest overall estimated impact scores due to the size and relative value of the cattle sector in Scotland. Pig diseases, by contrast, had lower overall impact, given the relatively small pig population in Scotland, estimated at 320,000. Hence the sector-specific impact of FMD and ASF in pigs and AHS in horses is high, but when, in this model framework, the impact is adjusted for the size of the Scottish populations, the overall estimated impact is low due to the small size of the populations of these species. The estimated impact of BTV in cattle is low when population size is not considered because the disease impacts of BTV in cattle are typically relatively mild, although cattle may act as a reservoir species (24) (Figure 1).

Multi-host diseases were assessed individually for each potentially affected sector. However, a real-world incursion would probably impact on all sectors. So for example, FMD has a high estimated impact in cattle alone, but when sheep and pigs are also considered, the estimated impact of FMD considerably outweighs that of all other diseases.

At the time of writing, the diseases with the highest potential impact and highest risk of incursion were BTV (sheep), FMD (cattle), HPAI and ND (Figure 1). This is largely due to the extent to which these pathogens were circulating in Western Europe and as the fact that the generic model for incursion weights imports of live animals and the vector or wildlife pathways most highly. ASF is increasing in terms of risk of introduction as it spreads in Western Europe, but the impact remains low due to the small pig population in Scotland. AHS is a disease with an impact that is similar to ASF but with a negligible chance of introduction, due to it being restricted to Sub Saharan Africa, in countries with no direct trade links to the UK (25). However, the global pattern of livestock diseases is constantly changing; LSD and PPR are good examples of diseases that until recently had never been reported in Europe (7–9). In addition, the global distribution of disease vectors is changing, for instance, *Aedes albopictus* larvae were found in Southern England for the first time in 2016 (26). Vector distributions are factored into the model, but a changing distribution of vectors could change the estimated impacts of vector borne diseases.

The relative chance of disease introduction changes with time, particularly as the global distribution of diseases changes or as disease regulations change. There are also seasonal variations associated with vector borne diseases or with annual variations in bird migrations. Whilst the chance of introduction is quite dynamic, the risks arising from the diseases are quite static, changes only result from changes to the size of the population at risk, or possibly to changes in our understanding of the pathogenicity of the disease.

The matrix emphasizes the importance of focusing on species for which Scotland has the largest populations. Whilst swine diseases are very high impact, they are less prominent in the matrix than cattle and sheep diseases for which Scotland has a very large population. The matrix also emphasizes how the same disease can affect different sectors in different ways. This particularly applies to FMD in cattle relative to sheep or pigs.

## Conclusion

We have presented a simple model framework that can be used to explore the interplay of the chance of disease incursion and the likely disease impact: the two components of risk assessment. The framework allows users to prioritize and assign risks to individual diseases. We have demonstrated that the outcomes are sensitive to purely local considerations such as the balance of species in the livestock population. The model focuses the relative impacts of different diseases beyond the individual animal or farm and compares populations as a whole.

## Data Availability Statement

All datasets generated for this study are included in the article/Supplementary Material.

## Author Contributions

PB, IM, BB, HA, and LB contributed conception and design of the model. HR contributed the risk of incursion database. All authors reviewed the manuscript.

## Conflict of Interest

The authors declare that the research was conducted in the absence of any commercial or financial relationships that could be construed as a potential conflict of interest. The reviewer FM declared a past co-authorship with one of the author HA to the handling Editor.
