# Supplementary material for: A Tool for Prioritizing Livestock Disease Threats to Scotland
Source: Front Vet Sci. 2020 Apr 24;7:223. doi: 10.3389/fvets.2020.00223 (PMC7193530; doi:10.3389/fvets.2020.00223)
Supplement: Supplementary file 1 [file Data_Sheet_1.docx]

**Supplementary information for: A tool for prioritising livestock disease threats to Scotland**

PR Bessell, HK Auty, H Roberts, IJ McKendrick, BMC Bronsvoort, LA Boden

| **Parameter** | **Factor** | **Description of Scale** | **Score range** |
| --- | --- | --- | --- |
| Propensity for spread | Rate of spread within premises | Very slow (typically months between transmission events) = 1  Very rapid spread = 4 | 1 - 4 |
|  | Rate of spread between premises | Very slow spread – typically only through animal movements = 1  Disease is spread very quickly, by biological vectors = 4 | 1 - 4 |
|  | Potential for silent spread | Signs of infection are easily recognised and present early = 1  The presence of disease will be concealed for months = 4 | 1 - 4 |
|  | Are the conditions for spread present in Scotland (vectors etc) | Conditions for spread are not believed to be present = 1  Disease can spread all year = 4 | 1 - 4 |
| Mitigation factors | Vaccines | No vaccine = 0  Effective vaccines = 2 | 0 – 2 |
|  | Wildlife reservoir | Widespread reservoir species = 0  No reservoir species = 4 | 0 - 4 |
|  | Vector reservoir | Biological vector = 0  No vector = 4 | 0 - 4 |
|  | Control through biosecurity | Biosecurity has no impact on controlling disease = 0  Biosecurity will control the disease = 4 | 0 - 4 |
|  | Control through movement bans | Movement bans have no impact on controlling disease = 0  Movement bans will control the disease = 4 | 0 - 4 |

Table S1. The scoring criteria applied to the parameters for extent of spread of the disease that were used in this impact model.

| **Parameter** | **Factor** | **Description** | **Score range** |
| --- | --- | --- | --- |
| Animal welfare and production | Animal mortality | No mortality = 0  All infected animals will die = 4 | 0 - 4 |
|  | Morbidity – severity of disease in animals | No morbidity / impairment = 0  Severe permanent impairment = 4 | 0 – 4 |
|  | Duration of infection / length of morbidity | Very brief infection 1 – 2 days = 1  Chronic infection = 4 | 1 – 4 |
|  | Impact of disease on production | Production is not impaired = 0  Production is permanently reduced = 4 | 0 – 4 |
| Human health risk | Morbidity / severity of disease in humans | No disease in humans = 0  Infection causes sever disease in humans = 2 | 0 – 2 |
|  | Potential for establishment | The disease could not become established in human populations = 0  The disease is very likely to become established in human populations = 2 | 0 – 2 |
|  | Likelihood of transmission to humans | The disease would not spill over to humans = 0  The disease is likely to spill over to humans = 2 | 0 – 2 |
|  | Potential for transmission between humans | The disease can not spread between humans = 0  The disease will spread easily between humans = 2 | 0 – 2 |

Table S2. The scoring criteria applied to the parameters for direct impact of the disease that were used in this impact model.

| **Parameter** | **Factor** | **Description** | **Score range** |
| --- | --- | --- | --- |
| Wider society | Potential cost to the exchequer | No cost = 0  High cost = 2 | 0 – 2 |
|  | Impact on the rural community | No impact = 0  High impact = 2 | 0 – 2 |
|  | Impact on the sector | No impact on other producers = 0  Other producers are severely impacted = 2 | 0 – 2 |
|  | Impact on rural usage | Rural usage is not impaired = 0  Severely impaired = 2 | 0 – 2 |
| International trade | Relative importance of export trade to the industry sector | There are no exports = 0  Exports are of great importance to the sector = 3 | 0 - 3 |
|  | Resource and time required to re-establish intra-community trading status (demonstrating freedom from infection) | Not required = 0  Very difficult, slow and resource intensive = 3 | 0 - 3 |
|  | Obligation to control under EU law | No obligation = 0  Obligatory control = 3 | 0 - 3 |
|  | Impact on trade | Trade is not impacted = 0  Trade is severely impacted = 3 | 0 - 3 |
|  | Potential for zoning | Zoning is possible at the farm level = 0  Little zoning possible = 3 | 0 - 3 |

Table S3. The scoring criteria applied to the parameters for indirect impact of the disease that were used in this impact model.


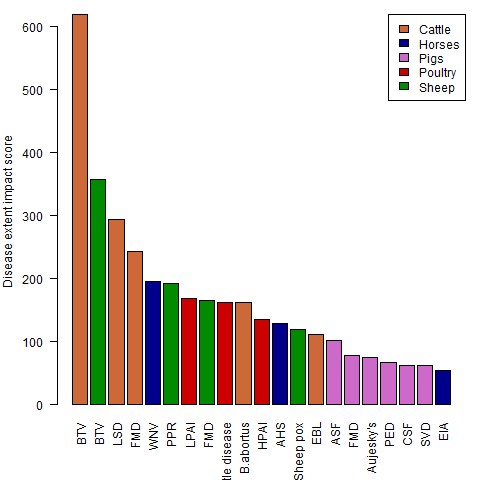


Figure S1. Barplot of the contribution to the impact scores from the extent of spread of the disease element (${Pop}_{i}^{T}\beta_{d}p_{d}$).


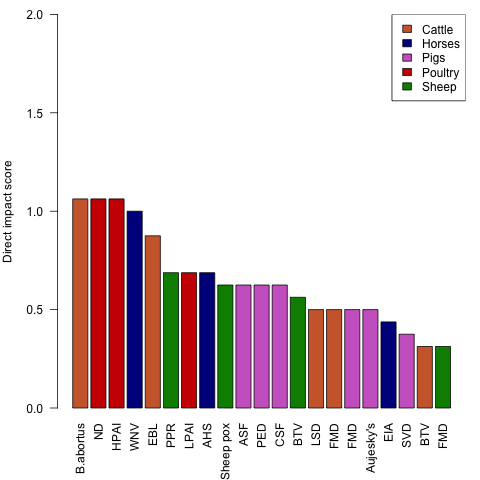


Figure S2. Barplot of the contribution to the impact scores from the direct impact of the disease element ($a_{d}+h_{d}$).


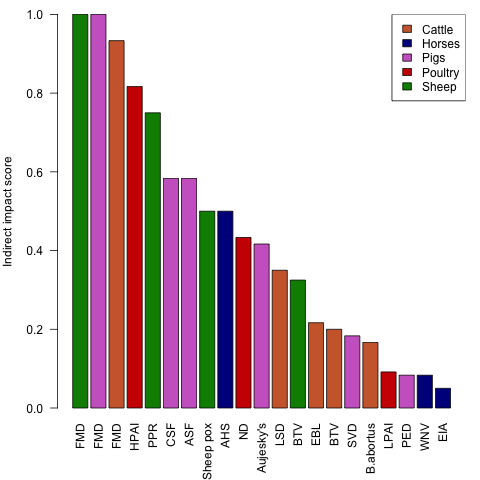


Figure S3. Barplot of the contribution to the impact scores from the indirect impacts of diseases element of the model ($c_{d}t_{d}$).


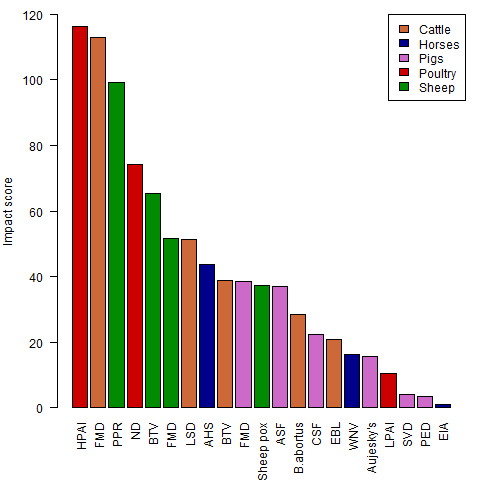


Figure S4. Final risk scores of the modelled diseases.


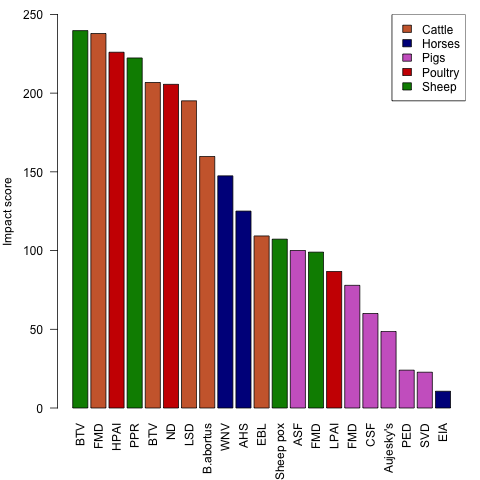


Figure S5. Risk score based on including the indirect impacts additively rather than multiplicatively.
