# Supplementary material for: A Tool for Prioritizing Livestock Disease Threats to Scotland
Source: Front Vet Sci. 2020 Apr 24;7:223. doi: 10.3389/fvets.2020.00223 (PMC7193530; doi:10.3389/fvets.2020.00223)
Supplement: Supplementary file 2 [file Data_Sheet_2.docx]

**Supplementary information for: A tool for prioritising livestock disease threats to Scotland**

PR Bessell, HK Auty, H Roberts, IJ McKendrick, BMC Bronsvoort, LA Boden

# African swine fever (ASF) example

The below example shows the process for calculating the impact of ASF based on the equation:

$$r_{di}={Pop}_{i}^{T}\beta_{di}{(1-p}_{di})(a_{di}+h_{di})c_{di}t_{di}$$

## Population at risk

| **Parameter** | **Value** |
| --- | --- |
| Pig population of Scotland | 316,736 |
| Livestock units (LUs) / pig | 0.189 |
| Total LUs | 59,863 |
| Square root LUs (Pop^T^_i_) | 244.67 |

## Propensity for spread

| **Determining factor** | **Value / max value** |
| --- | --- |
| Rate of spread within premises | 4 / 4 |
| Rate of spread between premises | 3 / 4 |
| Potential for silent spread | 1 / 4 |
| Are the conditions for spread present in Scotland | 4 / 4 |
| Sum (determining factors) | 12 |
| Sum(max( determining factors)) | 16 |
| $\beta_{d}=$Sum (determining factors) /  Sum (max( determining factors)) | 0.75 |

## Mitigation factors

| **Determining factor** | **Value** |
| --- | --- |
| Are effective vaccines available | 0 / 2 |
| Is there a wildlife reservoir | 2 / 4 |
| Is there a vector reservoir | 2 / 4 |
| Can the disease be controlled through biosecurity | 2 / 4 |
| Can spread be controlled through movement bans | 2 / 4 |
| Sum (determining factors) | 8 |
| Sum (max( determining factors) | 18 |
| ${(1-p}_{d})=$1 - Sum (determining factors) /  Sum (max( determining factors)) | 0.556 |

## Animal welfare and production

| **Determining factor** | **Value** |
| --- | --- |
| Rate of mortality among infected animals | 3 / 4 |
| Severity of morbidity in infected animals | 3 / 4 |
| Duration of morbidity in infected animals | 1 / 4 |
| Impact of infection on production | 3 / 4 |
| Sum (determining factors) | 10 |
| Sum (max( determining factors)) | 16 |
| $a_{d}=$Sum (determining factors) /  Sum (max( determining factors)) | 0.625 |

## Human health risk

| **Determining factor** | **Value** |
| --- | --- |
| Morbidity / severity of disease in humans | 0 / 2 |
| Potential for establishment | 0 / 2 |
| Likelihood of transmission to humans | 0 / 2 |
| Potential for transmission between humans | 0 / 2 |
| Sum (determining factors) | 0 |
| Sum (max( determining factors)) | 8 |
| $h_{d}=$Sum (determining factors) /  Sum (max( determining factors)) | 0.0 |

## Impact on wider society

| **Determining factor** | **Value** |
| --- | --- |
| Potential cost to the exchequer | 2 / 2 |
| Impact on the rural community | 1 / 2 |
| Impact on the sector | 2 / 2 |
| Impact on rural usage | 0 / 2 |
| Sum (determining factors) | 5 |
| Sum (max( determining factors)) | 8 |
| $c_{d}=$Sum (determining factors) /  Sum (max( determining factors)) | 0.625 |

## Impact on international trade

| **Determining factor** | **Value** |
| --- | --- |
| Relative importance of export trade to the industry sector | 3 / 3 |
| Resource and time required to re-establish intra-community trading status (demonstrating freedom from infection) | 2 / 3 |
| Obligation to control under EU law | 3 / 3 |
| Impact on trade | 3 / 3 |
| Potential for zoning | 3 / 3 |
| Sum (determining factors) | 14 |
| Sum (max( determining factors)) | 15 |
| $t_{d}=$Sum (determining factors) /  Sum (max( determining factors)) | 0.933 |

## Total risk

$$r_{di}={Pop}_{i}^{T}\beta_{di}{(1-p}_{di})(a_{di}+h_{di})c_{di}t_{di}$$

R_ASF_ = 244.67 x 0.75 x 0.56 x 0.625 x 0.625 x 0.933

R_ASF_ = 37.2
